# Cytomegalovirus Antibodies and Coronary Artery Disease in People with HIV: A Cohort Study

**DOI:** 10.3390/v17020231

**Published:** 2025-02-07

**Authors:** Moises Alberto Suarez-Zdunek, Andreas Dehlbæk Knudsen, Andreas Fuchs, Nikolai Søren Kirkby, Thomas Benfield, Jan Gerstoft, Marius Trøseid, Sisse Rye Ostrowski, Lars Valeur Køber, Klaus Fuglsang Kofoed, Susanne Dam Nielsen

**Affiliations:** 1 Viroimmunology Research Unit, Department of Infectious Diseases, Copenhagen University Hospital—Rigshospitalet, 2100 Copenhagen, Denmark; 2 Department of Cardiology, Copenhagen University Hospital—Rigshospitalet, 2100 Copenhagen, Denmark; 3 Department of Clinical Microbiology, Copenhagen University Hospital—Rigshospitalet, 2100 Copenhagen, Denmark; 4 Department of Infectious Diseases, Copenhagen University Hospital—Amager and Hvidovre, 2650 Hvidovre, Denmark; 5 Department of Clinical Medicine, Faculty of Health and Medical Sciences, University of Copenhagen, 2200 Copenhagen, Denmark; 6 Department of Infectious Diseases, Copenhagen University Hospital—Rigshospitalet, 2100 Copenhagen, Denmark; 7 Research Institute of Internal Medicine, Oslo University Hospital Rikshospitalet, 0372 Oslo, Norway; 8 Section of Clinical Immunology and Infectious Diseases, Oslo University Hospital Rikshospitalet, 0372 Oslo, Norway; 9 Department of Clinical Immunology, Copenhagen University Hospital—Rigshospitalet, 2100 Copenhagen, Denmark

**Keywords:** HIV, cytomegalovirus, coronary artery disease

## Abstract

People with HIV (PWH) have a high risk of coronary artery disease (CAD). Cytomegalovirus (CMV) co-infection is very common in PWH, but little is known about its association with CAD. We aimed to investigate if CMV IgG serostatus and concentrations are associated with CAD in PWH. From the Copenhagen Comorbidity in HIV Infection (COCOMO) Study we included PWH with coronary CT angiography (CCTA) and quantitative CMV IgG concentration measurements. We measured the stenosis grades and plaque volumes in the coronary vessels. Using multivariable regressions adjusted for traditional CAD risk factors, we investigated if CMV IgG serostatus and concentrations were associated with any atherosclerosis, obstructive CAD, or plaque volumes. We included 620 PWH of whom 586 had positive CMV serostatus, which was not associated with any atherosclerosis, obstructive CAD, or plaque volumes. A doubling of CMV IgG concentrations was associated with any atherosclerosis (OR 1.21 [95% CI: 1.06–1.39]), obstructive CAD (OR 1.31 [95% CI: 1.07–1.59]), and higher total plaque volume (1.56 [95% CI: 1.21–2.01] fold increase), but the association did not remain significant after adjustment for traditional CAD risk factors. This indicates that CMV IgG serostatus and concentrations are not independently associated with prevalent CAD in PWH.

## 1. Introduction

People with HIV (PWH) have a substantially higher burden of comorbidities compared to the general population [1]. Diseases attributable to atherosclerosis, such as coronary artery disease (CAD), are common in PWH [2,3], and living with HIV is associated with twice the risk of myocardial infarction and stroke [4].

Most PWH have been infected with cytomegalovirus (CMV) [5,6], which establishes life-long latency and may reactivate during periods of immunosuppression. A large meta-analysis showed that CMV seropositivity is associated with a 30% higher risk of mortality from cardiovascular diseases (CVD) in the general population [7], although results are conflicting [8]. Various mechanisms have been proposed, including increased systemic inflammation and endothelial dysfunction that promote coagulation, migration of atherogenic T cells, and uptake of low-density lipoprotein (LDL) by activated monocytes in atherosclerotic plaques [9]. These effects may be more pronounced in PWH who are already in a state of increased inflammation and immune activation, which can be reduced by treating CMV [10,11].

In a large study of PWH naïve to antiretroviral therapy (ART), CMV seropositivity doubled the hazard of vascular events, including myocardial infarction and stroke [12]. However, a recent study of well-treated PWH at low to moderate risk of CVD found no association between CMV immunoglobulin G (IgG) titres and the presence of coronary plaque [13]. Thus, there is a lack of consensus, and it is not known if CMV is associated with CAD in non-selected, well-treated PWH. If the association between CMV and vascular events can be substantiated by demonstrating an association between CMV and CAD, screening for CMV serostatus may help identify PWH in need of intensified cardiovascular prophylaxis.

We aimed to determine if CMV IgG serostatus and CMV IgG concentrations are associated with prevalent CAD in PWH. We additionally aimed to determine if markers of inflammation and endothelial dysfunction mediate the association between CMV IgG concentrations and prevalent CAD. We hypothesised that positive CMV IgG serostatus and higher CMV IgG concentrations are associated with prevalent CAD in PWH. We further hypothesised that markers of inflammation and endothelial dysfunction mediate this association.

## 2. Materials and Methods

### 2.1. Overview

The Copenhagen Comorbidity in HIV Infection (COCOMO) study is a prospective cohort study aiming to investigate comorbidities in PWH (ClinicalTrials.gov: NCT02382822). All PWH who were at least 18 years old and followed at the HIV outpatient clinics of Copenhagen University Hospital, Rigshospitalet, or Amager-Hvidovre were invited to participate between March 2015 and November 2016, as described previously [14]. We used baseline data from COCOMO and included all COCOMO participants with available research cardiac CT angiography (CCTA) of diagnostic quality and measured plasma CMV IgG concentrations.

### 2.2. Data Acquisition

At inclusion, all participants underwent physical examinations, including measurements of height, weight, and blood pressure. Non-fasting blood samples were collected for measurements of plasma concentrations of glycated haemoglobin (HbA1c), glucose, LDL, creatinine, and high-sensitivity C-reactive protein (hsCRP). Plasma was stored at −80 °C and thawed for measurements of CMV IgG concentrations using the LIAISON^®^ CMV IgG II indirect chemiluminescent immunoassay (DiaSorin, Saluggia, Italy [15]) with a range of detectability between 5.0 and 180 U/mL. Samples with concentrations above this range were diluted up to 10 times to achieve measurable concentrations. Concentrations of interleukin 6 (IL-6), tumour necrosis factor alpha (TNF-α), soluble thrombomodulin (sTM), and syndecan-1 were measured using Luminex^®^ Multiplex Assays (R&D systems, Minneapolis, MN, USA).

Participants filled out a questionnaire on demographics, lifestyle, and medical history. Patient records were reviewed for time with HIV, HIV RNA, current CD4+ and CD8+ T cell counts, CD4+ T cell nadir, previous CDC stage 3-defining opportunistic illnesses [16], use of ART, as well as hepatitis C virus (HCV) antibody tests.

### 2.3. Coronary CT Angiography

COCOMO participants with an estimated glomerular filtration rate (eGFR) ≥ 60 mL/min/1.73 m^2^ were offered a CCTA using a 320 slice (thickness of 0.5 mm) multi-detector CT scanner (Aquilion ONE ViSION Edition, Canon Medical Systems, Otawara, Japan). Based on heart rate, oral metoprolol or ivabradine was administered prior to the CCTA as previously described [3]. Blinded of participant information, coronary artery branches were divided into 17 segments, and vessels with a diameter of ≥1.5 mm were analysed for the presence and degree of luminal stenoses [17,18]. In segments with atherosclerosis, the volume and composition of atherosclerotic lesions were recorded semi-automatically with dedicated software (QAngio^®^ CT Research Edition version 2.02, Medis Medical Imaging, Leiden, The Netherlands). Plaque composition analyses included the calculation of dense calcium, fibrotic, fibro-fatty, and necrotic core components based on attenuation gradients [19].

### 2.4. Definitions

We defined positive CMV IgG serostatus as a CMV IgG concentration ≥14 U/mL, as defined by the manufacturer. Any CAD and obstructive CAD were defined as a maximum luminal stenosis ≥1% and ≥50%, respectively. Extensive CAD was defined as any atherosclerosis involving >5 of 17 coronary segments [17]. Total plaque volume was calculated as the sum of plaque volumes in all segments with atherosclerosis. Calcified and fibrotic plaque volumes were defined as the sums of volumes of all dense calcium plaques and fibrotic plaques, respectively. Inflamed plaque volume was defined as the sum of volumes of all fibro-fatty and necrotic core plaques.

Hypertension was defined as systolic blood pressure ≥140 mmHg and/or diastolic blood pressure ≥90 mmHg and/or the use of antihypertensive medications. Diabetes mellitus was defined as non-fasting plasma glucose ≥11.1 mmol/L and/or HbA1c ≥6.5%. Dyslipidaemia was defined as LDL ≥3.0 mmol/L and/or the use of lipid-lowering medications. Smoking was self-reported. Body mass index (BMI) in kg/m^2^ was categorised as underweight (<18.5), normal (18.5–24.9), overweight (25.0–29.9), and obese (≥30.0).

### 2.5. Statistical Analyses

For all analyses, the exposure variables were CMV IgG serostatus and CMV IgG concentrations. We defined three adjusted models: model 1 (adjusted for age, sex, and smoking), model 2 (model 1 adjusted for diabetes mellitus and dyslipidaemia), and model 3 (model 1 adjusted for current CD4+ T cell count). A directed acyclic graph of the adjusted models is provided in Supplementary Figure S1 [20].

For primary analyses, we used binary outcomes of any atherosclerosis and obstructive CAD, as well as a continuous outcome of total plaque volume (µL). For binary outcomes, we used logistic regressions to calculate odds ratios (OR) of exposure variables before and after adjustment for models 1–3. For total plaque volume, we used linear regressions to calculate regression coefficients of exposure variables before and after adjustment for models 1–3, and the effects were presented as a fold increase in total plaque volume. In a post hoc sensitivity analysis, we stratified the results by CD4+ T cell nadir. Furthermore, to account for a potential impact of time since HIV infection, we performed two additional post hoc sensitivity analyses. First, we investigated the interaction between time since HIV infection and CMV IgG concentrations. Second, we employed a case–control design where participants with any atherosclerosis were classified as cases and were matched 1:1 with participants without any atherosclerosis on time since HIV infection (difference of maximum 5 years). Using conditional logistic regressions adjusted for models 1–3, we calculated ORs of CMV IgG serostatus and CMV IgG concentrations.

We planned mediation analyses of markers of inflammation (hsCRP, IL-6, and TNF-α) and endothelial dysfunction (sTM and syndecan-1) to be performed only in the presence of an association between CMV IgG concentrations and total plaque volume, as well as CMV IgG concentrations and the marker of inflammation or endothelial dysfunction [21]. Using the R *mediation* package, we computed the mediation effects of concentrations of hsCPR and IL-6 with bootstrapped confidence intervals (CI) using bias-corrected accelerated percentile limits with 1000 simulations [22]. In post hoc sensitivity analyses, we estimated the effect of age using the same method and repeated the primary analyses adjusting for age only.

For prespecified exploratory analyses, we investigated continuous outcomes of calcified, fibrotic, and inflamed plaque volumes and a binary outcome of extensive CAD.

To meet the linearity assumption in analyses of plaque volumes, volumes of 0 µL were replaced with 0.1 µL, and all values were log-transformed. In a prespecified sensitivity analysis, we analysed the original total plaque volumes using zero-inflated negative binomial regression.

Two-sided *p*-values < 0.050 were considered significant. For all analyses, we used R (version 4.3.2, R Foundation for Statistical Computing, Vienna, Austria).

## 3. Results

A total of 620 participants had a diagnostic CCTA and measured CMV IgG concentrations. Of the included participants, 89% were male, and the median age was 50 years. Almost all were treated with ART and had undetectable HIV RNA, and 95% had positive CMV IgG serostatus (Table 1). Any atherosclerosis was present in 45% of participants (Table 2) [3].

### 3.1. Associations with CAD

**Figure 1:**
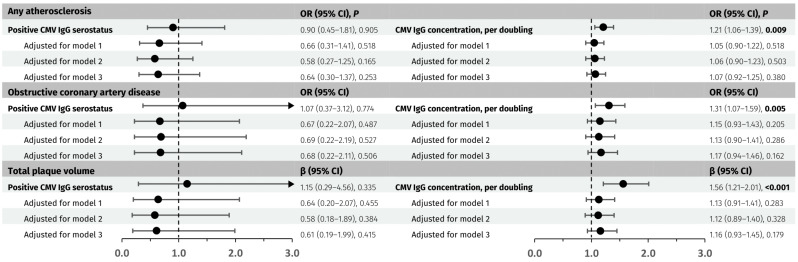
Associations of cytomegalovirus antibodies with coronary artery disease. CMV, cytomegalovirus. Model 1: age, sex, and smoking. Model 2: age, sex, smoking, diabetes mellitus, and dyslipidaemia. Model 3: age, sex, smoking, and current CD4+ T cell count. OR, odds ratio. CI, confidence interval. β, regression coefficient to be interpreted as a fold increase in total plaque volume. The circles represent OR for any atherosclerosis and obstructive coronary artery disease, or β for total plaque volumes, respectively, with the horizontal lines representing the associated 95% CI.

Positive CMV IgG serostatus was not associated with any atherosclerosis (OR 0.90 [95% CI: 0.45–1.81], *p* = 0.774), obstructive CAD (OR 1.07 [95% CI: 0.37–3.12], *p* = 0.905), or total plaque volume (1.15 [95% CI: 0.29–4.56] fold increase, *p* = 0.847) before or after adjustment for models 1, 2, and 3 (Figure 1).

However, each doubling of CMV IgG concentrations was associated with any atherosclerosis (OR 1.31 [95% CI: 1.07–1.59], *p* = 0.009), obstructive CAD (OR 1.21 [95% CI: 1.06–1.39], *p* = 0.005), and 1.56 (95% CI: 1.21–2.01, *p* < 0.001) fold higher total plaque volume. None of these associations remained significant after adjustment for age, sex, and smoking (model 1), or after further adjustment for variables from models 2–3 (Figure 1).

### 3.2. Associations with Plaque Composition and Extensive CAD

**Figure 2:**
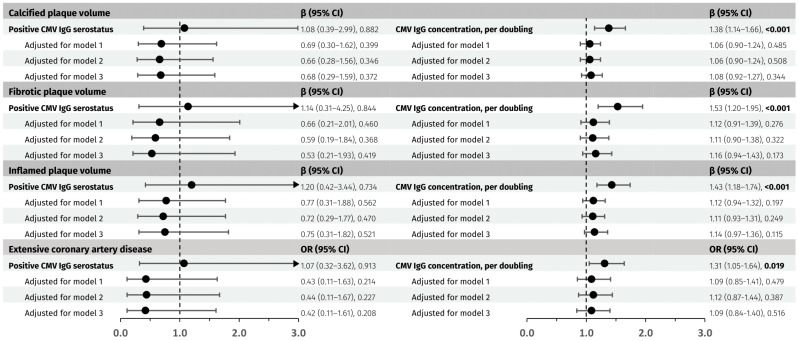
Associations of cytomegalovirus antibodies with plaque composition and extensive disease. CMV, cytomegalovirus. Model 1: age, sex, and smoking. Model 2: age, sex, smoking, diabetes mellitus, and dyslipidaemia. Model 3: age, sex, smoking, and current CD4+ T cell count. OR, odds ratio. CI, confidence interval. β, regression coefficient to be interpreted as a fold increase in total plaque volume. The circles represent OR for extensive coronary artery disease or β for plaque volumes, respectively, with the horizontal lines representing the associated 95% CI.

We found no association between positive CMV IgG serostatus and calcified, fibrotic, or inflamed plaque volumes, or extensive CAD before or after adjustments (Figure 2).

Each doubling of CMV IgG concentrations was associated with higher calcified plaque volume (1.38 [95% CI: 1.14–1.66] fold increase, *p* < 0.001), fibrotic plaque volume (1.53 [95% CI: 1.20–1.95] fold increase, *p* < 0.001), inflamed plaque volume (1.43 [95% CI: 1.18–1.74] fold increase, *p* < 0.001), and extensive CAD (OR 1.31 [95% CI: 1.05–1.64], *p* = 0.019). No associations remained significant after adjustment for either model 1, 2, or 3 (Figure 2).

### 3.3. Impact of Inflammation and Endothelial Dysfunction

Each doubling of hsCRP concentrations was associated with a 1.35 (95% CI: 1.12–1.64, *p* = 0.002)-fold higher total plaque volume. Likewise, the doubling of IL-6 concentrations was associated with a 1.81 (95% CI: 1.40–2.35, *p* < 0.001)-fold higher total plaque volume. Concentrations of TNF-α, sTM, and syndecan-1 were not associated with total plaque volume (Supplementary Table S1). Conversely, the doubling of CMV IgG concentrations was associated with higher concentrations of hsCRP (1.16 [95% CI: 1.07–1.25]-fold increase, *p* < 0.001), IL-6 (1.07 [95% CI: 1.01–1.13]-fold increase, *p* = 0.02), and TNF-α (1.07 [95% CI: 1.04–1.11]-fold increase, *p* < 0.001). The associations with hsCRP and TNF-α remained significant after adjustments (Supplementary Table S2). Overall, the prespecified conditions for mediation were only met for hsCRP and IL-6, so we only proceeded with mediation analyses for hsCRP and IL-6 concentrations. Higher hsCRP and IL-6 concentrations mediated 11% (*p* < 0.01) and 10% (*p* = 0.038) of the total effect of CMV IgG concentrations on total plaque, respectively. After conditioning the analyses for increasing age, no mediation effect was present for either hsCRP or IL-6 concentrations.

### 3.4. Sensitivity Analyses

In a sensitivity analysis only adjusting for age, doubling of CMV IgG concentrations was not associated with either any atherosclerosis (OR 1.08 [95% CI: 0.93–1.25], *p* = 0.330), obstructive CAD (OR 1.18 [95% CI: 0.95–1.45], *p* = 0.131), or total plaque volume (1.19 [95% CI: 0.95–1.49] fold increase, *p* = 0.124). Accordingly, age accounted for 69% (*p* = 0.002) of the effect of CMV IgG concentrations on total plaque volume adjusted for model l. Using the zero-inflated negative binomial regression did not change our findings. Consistently, among participants with CD4+ T cell nadir <200 cells/µL, there were no significant findings for CMV IgG serostatus.

We did not find significant interactions between CMV IgG concentrations and time with HIV infection on the association with any atherosclerosis, obstructive CAD, or total plaque volume (all *p* for interaction > 0.16). Matching participants with any atherosclerosis with participants without any atherosclerosis on time since HIV infection yielded consistent results, as CMV IgG serostatus (all *p* > 0.38) and CMV IgG concentrations (all *p* > 0.28) were not associated with any atherosclerosis after adjustment for models 1, 2, or 3.

## 4. Discussion

In this study of a large cohort of well-treated PWH not selected on their a priori CVD risk, we found a strong association between higher CMV IgG concentrations and CAD across different severities of CAD and plaque phenotypes. However, this association was not independent of age and other traditional CVD confounders.

In our cohort, around 95% of PWH had positive CMV IgG serostatus. This is much higher than the 39–48% estimated for the general population in Western countries [23], but consistent with studies of PWH in Europe [10]. We found no association between CMV serostatus and CAD, regardless of stenosis grade or plaque phenotype. One previous cardiac CT study of 78 PWH without CVD reported no association between CMV IgG serostatus and presence of plaque, but only 7 participants had negative serostatus. Two additional studies of PWH without CVD investigated CMV serostatus and CAD on cardiac CT, but due to few PWH with negative serostatus, the effect of CMV serostatus was not reported [13,24]. Despite markedly more CMV seronegative participants than in previous studies, the power to preclude an association between positive CMV serostatus and CAD in our study was limited. However, most estimates of positive CMV serostatus pointed in the direction of lower odds of CAD and plaque volumes, which supports that this may not be due to inadequate power but because CMV serostatus itself is not an ideal marker of prevalent CAD in PWH.

We found an association between higher CMV IgG concentrations and CAD, but this effect was not independent of traditional CAD risk factors. Accordingly, most of the effect was explained by confounding by age rather than the expected inflammation or endothelial dysfunction. The recent Randomized Trial to Prevent Vascular Events in HIV (REPRIEVE) study that included 672 PWH found no association between CMV IgG concentrations and any atherosclerosis or high Leaman scores on CCTA either before or after adjustments [13], but generalisability was limited due to the exclusion of participants with or at high risk of CVD. Two smaller studies of PWH excluding PWH with CVD found that CMV IgG concentrations were independently associated with coronary calcium scores [24,25], but lack of contrast enhancement precluded analysis of non-calcified plaque and CAD extent, which enables a more accurate assessment of the risk of myocardial infarction [17]. The lack of an independent association between CMV IgG concentrations and CAD in our non-selected cohort of PWH was surprising, as higher CMV IgG concentrations were associated with subclinical carotid atherosclerosis assessed by higher intima-media thickness or lower arterial distensibility independently of age [24,26,27]. Speculatively, CMV may exert atherogenic effects of differential magnitudes on varying vascular sites.

Additionally, we found that higher CMV IgG concentrations were associated with higher concentrations of hsCRP, IL-6, and TNF-α, and the associations remained significant for hsCRP and TNF-α after adjustment for traditional cardiovascular risk factors. Few studies of PWH have investigated the associations between CMV IgG concentrations and inflammatory markers and found positive correlations with hsCRP and IL-6, but not consistently after adjustments [13,28]. However, the finding of an association between CMV IgG concentrations and inflammatory markers after adjustment for confounders is in line with studies from people without HIV and supports a potential pathway between CMV and atherosclerotic CVD in PWH [29,30,31]. The cross-sectional design cannot establish the direction of the association between CMV IgG concentrations and inflammation, but since interaction between HIV and CMV has been hypothesised to synergistically promote proatherogenic inflammation [32], an effect of CMV may become more pronounced with a long time since HIV infection. As primary CMV infection is most commonly asymptomatic, time since primary CMV infection was not possible to assess, but in post hoc exploratory analyses, we found no interaction between time since HIV infection and CMV IgG serostatus and CMV IgG concentrations.

Higher CMV IgG concentrations are often interpreted as an indicator of higher subclinical CMV activity because higher CMV IgG concentrations are associated with a higher proportion of CMV-specific memory T cells in PWH [33] and with the severity of CMV reactivations in kidney transplant recipients [34]. Furthermore, CMV IgG is readily measurable, in contrast to plasma CMV DNA, a detectable level of which is not expected in immunocompetent, well-treated PWH. However, antibody concentrations are not ideal surrogates of CMV-specific cellular activation. As much as 20% of all CD8+ T cells may be CMV-specific in PWH [35], and they often express chemokine and costimulatory receptors that correspond to chemokines produced by vascular endothelium when stimulated by CMV [36]. Accordingly, CMV-specific T cells and their responses were associated with carotid atherosclerosis [37]. Investigating if CMV-specific T cells are associated with subclinical CAD using CCTA may provide additional insight into CAD pathogenesis in PWH.

The fact that we found no independent association between CMV and CAD contrasts with a study of 6111 ART-naïve PWH, which found that CMV seropositivity doubled the hazard of cardiovascular and cerebrovascular events independently of age, sex, and infection-related risk factors [12]. These findings, however, are difficult to compare as results from ART-naïve PWH are difficult to extrapolate to well-treated PWH, and our outcomes included subclinical CAD at earlier stages prior to plaque rupture. Furthermore, we did not include non-coronary atherosclerosis.

An important strength of this study is the large sample of well-characterised PWH, and this is the first study of the role of CMV on subclinical CAD in PWH who are not selected on their prior CVD risk. Because most participants had positive CMV IgG serostatus, we had excellent power to investigate the association between CMV IgG concentrations and CAD. However, reflecting the population of PWH in Denmark, most participants were male, and this limits generalisability to women with HIV. Last, the cross-sectional design cannot establish the direction of any causal paths, and follow-up studies with repeated CCTAs are necessary to assess the role of CMV on CAD progression.

## 5. Conclusions

The association between CMV IgG concentrations and prevalent CAD in PWH seemed to be attributable to traditional cardiovascular risk factors, most importantly higher age. As such, CMV IgG serostatus and concentrations were not independently associated with prevalent CAD in PWH. Longitudinal studies are necessary to determine if CMV increases the risk of de novo CAD and plaque progression in PWH.

## Figures and Tables

**Table 1:** Participant characteristics.

Demographics	
Age, years, median (IQR)	50.1 (42.8–57.1)
Male sex, *n* (%)	553 (89.2%)
Caucasian ethnicity, *n* (%)	536 (86%)
**Cardiovascular disease risk factors**	
Hypertension at baseline, *n* (%)	256 (41.3%)
Diabetes at baseline, *n* (%)	17 (2.7%)
Smoking at baseline, *n* (%)	
Current	177 (28.5%)
Former	220 (35.5%)
Never	223 (36.0%)
Body mass index, kg/m^2^, median (IQR)	24.3 (22.3–26.8)
Underweight, *n* (%)	17 (2.7%)
Normal, *n* (%)	345 (55.6%)
Overweight, *n* (%)	217 (35.0%)
Obese, *n* (%)	39 (6.3%)
Dyslipidaemia at baseline, *n* (%)	118 (19.0%)
Current use of lipid-lowering drugs	70 (59.3%)
**Infection-related characteristics**	
Positive CMV IgG serostatus, *n* (%)	586 (94.5%)
CMV IgG concentration, U/mL, median (IQR)	149 (114–473)
Time since HIV infection, years, median (IQR)	12.8 (6.0–20.7)
Current CD4+ T cell count <350 cells/µL, n (%)	40 (6.5%)
CD4+ T cell nadir <200 cells/µL, *n* (%)	235 (37.9%)
CD4+:CD8+ T cell ratio, median (IQR)	0.81 (0.58–1.14)
HIV RNA ≥ 50 copies/mL, *n* (%)	29 (4.7%)
Ever AIDS-defining condition, *n* (%)	109 (17.6%)
CMV disease	4 (0.7%)
Current use of ART, *n* (%)	612 (98.7%)
NRTI, *n* (%)	590 (95.2%)
NNRTI, *n* (%)	307 (49.5%)
Protease inhibitors, *n* (%)	176 (28.4%)
INSTI, *n* (%)	180 (29.0%)
Duration of ART, years, median (IQR)	9.8 (4.7–16.8)
Positive HCV antibody test, *n* (%)	71 (11.5%)

ART: antiretroviral therapy. Body mass index: underweight (<18.5 kg/m^2^), normal (18.5–24.9 kg/m^2^), overweight (25.0–29.9 kg/m^2^), and obese (≥30.0 kg/m^2^). HCV, hepatitis C virus. INSTI, integrase strand transfer inhibitor. IQR, inter-quartile ratio. NNRTI, non-nucleoside/nucleotide reverse transcriptase inhibitor. NRTI, nucleoside reverse transcriptase inhibitor.

**Table 2:** Cytomegalovirus IgG serostatus and coronary artery disease.

	Total (*N* = 620)	Positive CMV IgG Serostatus (*n* = 586)	Negative CMV IgG Serostatus (*n* = 34)
Coronary artery disease			
Any atherosclerosis	277 (44.7%)	261 (44.5%)	16 (47.1%)
Obstructive CAD	77 (12.4%)	73 (11.5%)	4 (11.8%)
Extensive CAD	58 (9.4%)	55 (9.4%)	3 (8.8%)
Total plaque volume in µL, mean, median (IQR)	142, 0 (0–153)	145, 0 (0–156)	90, 0 (0–106)
Among those with any plaque ^a^	315, 175 (86–374)	322, 180 (88–393)	200, 126 (67–278)
Calcified plaque volume in µL, mean, median (IQR)	30, 0 (0–9.6)	30, 0 (0–9.9)	20, 0 (0–5.5)
Among those with any plaque ^a^	66, 14 (2–52)	67, 14 (2–54)	43, 9 (2–37)
Fibrotic plaque volume in µL, mean, median (IQR)	91, 0 (0–114)	93, 0 (0–115)	58, 0 (0–89)
Among those with any plaque ^a^	202, 132 (66–253)	206, 133 (66–254)	128, 108 (50–201)
Inflamed plaque volume in µL, mean, median (IQR)	21, 0 (0–25)	21, 0 (0–28)	13, 0 (0–14)
Among those with any plaque ^a^	47, 31 (13–57)	47, 32 (14–58)	28, 18 (9–42)

CAD, coronary artery disease. IQR, inter-quartile ratio. ^a^ Defined as total plaque volume > 0 µL.

## Data Availability

The datasets generated and analysed for this study are not publicly available as they contain information that could compromise participant privacy and consent but are available from the corresponding author on reasonable request to the extent allowed by data protection legislation.

## Supplementary Materials

The following supporting information can be downloaded at https://www.mdpi.com/article/10.3390/v17020231/s1, Table S1: Analyses of markers of inflammation and endothelial dysfunction and total plaque volume; Table S2: Associations between CMV IgG concentrations and markers of inflammation and endothelial dysfunction; Figure S1: Directed acyclic graph of associations between investigated variables.

## Presentations of the data

Preliminary results from this study were presented in part as a poster at HIV Drug Therapy Glasgow 2024, 10–13 November 2024, Glasgow, Scotland, United Kingdom (abstract P293).
