# Supplementary material for: Cytomegalovirus Antibodies and Coronary Artery Disease in People with HIV: A Cohort Study
Source: Viruses. 2025 Feb 7;17(2):231. doi: 10.3390/v17020231 (PMC11860406; doi:10.3390/v17020231)
Supplement: Supplementary file 1 [file viruses-17-00231-s001.zip › viruses-3456769-supplementary-Figure S1.pdf]

SUPPLEMENTARY FIGURE S1

Directed acyclic graph of associations between investigated variables

Figure S1 Directed acyclic graph of associations between investigated variables

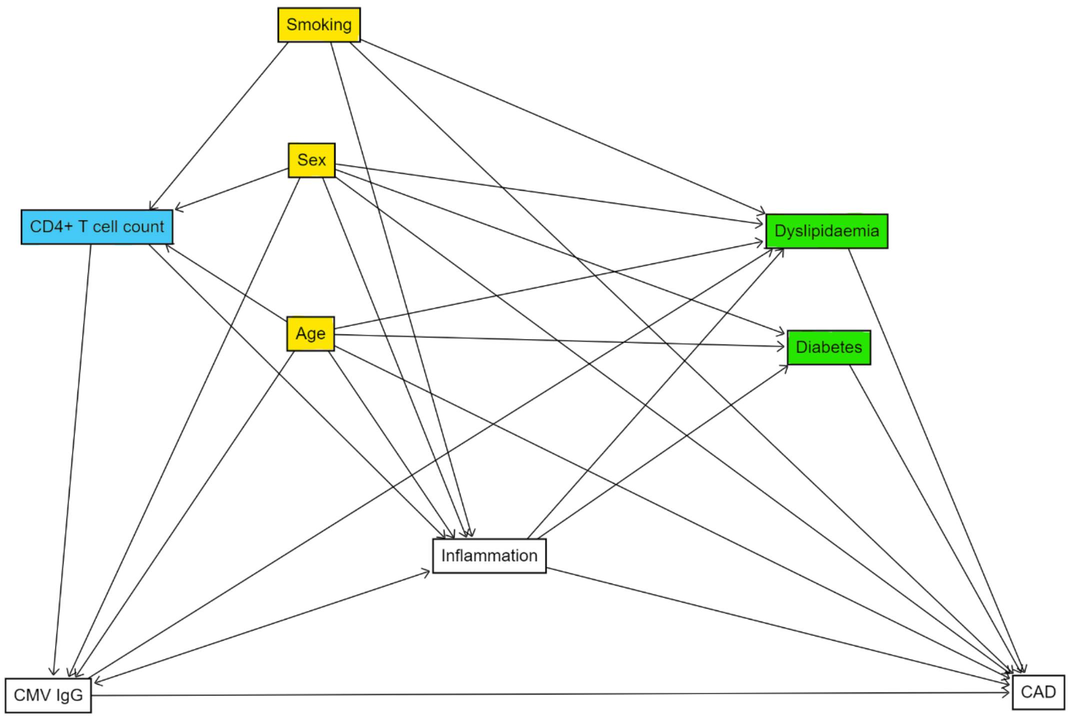

CMV IgG, cytomegalovirus immunoglobulin G. **CAD**, coronary artery disease.

CMV IgG serostatus and concentrations were hypothesised to be associated with CAD, in part mediated by inflammation. To investigate the total effect of CMV IgG serostatus and concentrations on CAD, adjusted models were designed and included: Model 1 (yellow) adjusting CMV IgG serostatus and concentrations for age, sex, and smoking; Model 2 (yellow and green) adjusting for model 1 and dyslipidaemia and diabetes; Model 3 (yellow and blue) adjusting for model 1 and current CD4+ T cell count. Mediation analyses were performed to investigate the effect of CMV IgG mediated by inflammation (indirect effect).

Created using DAGitty (Textor J et al. Int J Epidemiol 2016;45:1887-1894)
