# Supplementary material for: Cytomegalovirus Antibodies and Coronary Artery Disease in People with HIV: A Cohort Study
Source: Viruses. 2025 Feb 7;17(2):231. doi: 10.3390/v17020231 (PMC11860406; doi:10.3390/v17020231)
Supplement: Supplementary file 1 [file viruses-17-00231-s001.zip › viruses-3456769-supplementary-Table S1.pdf]

SUPPLEMENTARY TABLE S1

Analyses of markers of inflammation and endothelial dysfunction and total plaque volume

Table S1 Association of markers of inflammation and endothelial dysfunction and total plaque volume

	**β (95% CI), *P***	**aβ[1] (95% CI), *P***	**aβ[2] (95% CI), *P***	**aβ[3] (95% CI), *P***
hsCRP, per doubling	1.35 (1.12-1.64) **0.002**	1.06 (0.90-1.26) 0.464	1.06 (0.90-1.25) 0.495	1.05 (0.89-1.25) 0.516
IL-6, per doubling	1.81 (1.40-2.35) **<0.001**	1.19 (0.95-1.50) 0.136	1.24 (0.98-1.57) 0.074	1.18 (0.94-1.49) 0.163
TNF-α, per 1 pg/ml increase	1.07 (0.98-1.18) 0.122	1.01 (0.93-1.09) 0.889	1.01 (0.93-1.09) 0.807	1.00 (0.93-1.09) 0.914
sTM, per doubling	1.45 (0.70-3.02) 0.317	1.17 (0.62-2.19) 0.627	1.10 (0.59-2.08) 0.758	1.18 (0.63-2.21), 0.613
Syndecan-1, per doubling	1.01 (0.57-1.82), 0.962	0.71 (0.32-1.58), 0.396	0.70 (0.31-1.61), 0.404	0.63 (0.28-1.41), 0.257

hsCRP, high sensitivity C-reactive protein concentration. **IL-6**, interleukin 6 concentration. **TNF-α**, tumour necrosis factor alpha concentration. **sTM**, soluble thrombomodulin concentration. **Syndecan-1**, syndecan-1 concentration. **β**, regression coefficient to be interpreted as fold increase in total plaque volume. **aβ**, adjusted β. **CI**, confidence interval. [1]adjusted for age, sex, and smoking. [2]adjusted for age, sex, smoking, dyslipidaemia, and diabetes mellitus. [3]adjusted for age, sex, smoking, and current CD4+ T cell count.
