# Supplementary material for: Cytomegalovirus Antibodies and Coronary Artery Disease in People with HIV: A Cohort Study
Source: Viruses. 2025 Feb 7;17(2):231. doi: 10.3390/v17020231 (PMC11860406; doi:10.3390/v17020231)
Supplement: Supplementary file 1 [file viruses-17-00231-s001.zip › viruses-3456769-supplementary-Table S2.pdf]

SUPPLEMENTARY TABLE S2

Associations between CMV IgG concentrations and markers of inflammation and endothelial dysfunction

Table S2 Associations between CMV IgG concentrations and markers of inflammation and endothelial dysfunction

**Outcome variable**	**β (95% CI), *P***	**aβ[1] (95% CI), *P***	**aβ[2] (95% CI), *P***	**aβ[3] (95% CI), *P***
hsCRP, fold increase	1.16 (1.07-1.25), **<0.001**	1.12 (1.04-1.20), **<0.01**	1.12 (1.03-1.20), **<0.01**	1.12 (1.04-1.21), **<0.01**
IL-6, fold increase	1.07 (1.01-1.13), **0.02**	1.02 (0.97-1.08), 0.38	1.03 (0.98-1.09), 0.22	1.03 (0.97-1.09), 0.30
TNF-α, fold increase	1.07 (1.04-1.11), **<0.001**	1.06 (1.03-1.10), **<0.001**	1.07 (1.03-1.11), **<0.001**	1.07 (1.03-1.11), **<0.001**
sTM, fold increase	1.01 (1.00-1.03), 0.13	1.01 (0.99-1.03), 0.20	1.01 (0.99-1.03), 0.33	1.01 (0.99-1.03), 0.19
Syndecan-1, fold increase	1.00 (0.99-1.02), 0.483	1.00 (0.99-1.02), 0.64	1.00 (0.99-1.02), 0.76	1.01 (0.99-1.02), 0.51

CMV, cytomegalovirus. **hsCRP**, high sensitivity C-reactive protein concentration. **IL-6**, interleukin 6 concentration. **TNF-α**, tumour necrosis factor alpha concentration. **sTM**, soluble thrombomodulin concentration. **Syndecan-1**, syndecan-1 concentration. **β**, regression coefficient to be interpreted as fold increase in outcome variable per doubling of CMV IgG concentration. **aβ**, adjusted β. **CI**, confidence interval. [1]adjusted for age, sex, and smoking. [2]adjusted for age, sex, smoking, dyslipidaemia, and diabetes mellitus. [3]adjusted for age, sex, smoking, and current CD4+ T cell count.
